# Molecular Subtypes Recognition of Breast Cancer in Dynamic Contrast-Enhanced Breast Magnetic Resonance Imaging Phenotypes from Radiomics Data

**DOI:** 10.1155/2019/6978650

**Published:** 2019-10-30

**Authors:** Wei Li, Kun Yu, Chaolu Feng, Dazhe Zhao

**Affiliations:** ^1^Key Laboratory of Intelligent Computing in Medical Image (MIIC), Northeastern University, Ministry of Education, Shenyang, China; ^2^Biomedical and Information Engineering School, Northeastern University, Shenyang, China

## Abstract

**Background and Objective:**

Breast cancer is a major cause of mortality among women if not treated in early stages. Recognizing molecular markers from DCE-MRI directly to distinguish the four molecular subtypes without invasive biopsy is helpful for guiding treatment plans for breast cancer, which provides a fast way to consequential treatment plan decision in early time and best opportunity for patients.

**Methods:**

This study presents an approach of molecular subtypes recognition from breast cancer image phenotypes by radiomics. An improved region growth algorithm with dynamic threshold without user interaction is proposed for cancer lesion segmentation, which gives the precise border of lesion other than area with background. The lesions are extracted automatically based on radiologists' annotation which guarantees the lesion is segmented correctly. Various features are extracted on lesions data including texture, morphology, dynamic kinetics, and statistics features carried out on a large patient cohort, which are used to validate the relationship between image phenotypes and the molecular subtypes. A new algorithm of multimodel-based recursive feature elimination is applied on the radiomics data generated by the feature extraction process. This method obtains the feature subset with stable performance for different classification models, and the gradient boosting decision tree model gets the best results of both classification performance and imbalance performance on molecular subtypes.

**Result:**

From the experimental results, 69 optimal features from 143 original features are found by the multimodel-based recursive feature elimination algorithms and the gradient boosting decision tree classifier obtains a good performance with accuracy 0.87, precise 0.88, recall 0.87, and F1-score 0.87. The dataset with 637 patients in this paper has serious imbalance problem on different molecular subtypes, and the the robust features that are generated by multimodel-based recursive feature eliminiation algorithm make the gradient boosting decision tree classifier have good behaviors. The recognition precision for the four molecular subtypes of luminal A, luminal B, HER-2, and basal-like are 0.91, 0.89, 0.83, and 0.87, respectively.

**Conclusions:**

The improved lesion segmentation method gives more precise lesion edge, which not only saves the time of automatic extraction of lesion region of interest without threshold setting for each case, but also prevents the segmentation error by manual and prejudice from different radiologists. The feature selection algorithm of multimodel-based recursive feature elimination has the ability to find robust and optimal features that distinguish the four molecular subtypes from image phenotypes. The gradient boosting decision tree classifier rather plays a main role in recognition than other models used in this paper.

## 1. Introduction

Breast cancer is a major cause of mortality among women if not treated in early stages. Early screening and diagnosis have a lot to do with the therapeutic effect of prognosis. For noninvasive diagnosis, different imaging modalities can be used, such as molybdenum target X-ray, MRI, Ultra-sound, etc. Dynamic contrast enhanced breast magnetic resonance imaging (DCE-MRI) is one of the best imaging techniques that provide temporal information about the kinetics of the contrast agent in suspicious lesions along with acceptable spatial resolution. Recognizing molecular markers from DCE-MRI is helpful for guiding treatment plans for breast cancer.

The four molecular subtypes of breast cancer are analyzed in this paper, including luminal A, luminal B, human epidermal growth factor receptor-2 over-expressing (HER-2), and basal-like. However, tumor heterogeneity in cancers has been observed at the histological and genetic levels, and increased levels of intratumor genetic heterogeneity have been reported to be associated with adverse clinical outcomes [1]. Breast tumor structure contains a high degree of heterogeneity. This heterogeneity has been correlated with the level of tumor response to neoadjuvant chemotherapy [2].

The use and role of medical imaging technologies in clinical oncology has greatly expanded from primarily a diagnostic tool to include a more central role in the context of individualized medicine over the past decade [3]. Radiomics refers to the extraction and analysis of large amounts of advanced quantitative imaging features with high throughput from medical images obtained with computed tomography, positron emission tomography, or magnetic resonance imaging [4]. Radiomic studies can be used to understand relationships between imaging characteristics of tumors, such as heterogeneity, and their genetic characteristics, phenotype, or expected treatment outcome [5]. These data are combined with other patient data and are mined with sophisticated bioinformatics tools to develop models that may potentially improve diagnostic, prognostic, and predictive accuracy [6].

The radiomics methodology can be divided into distinct process which consists of five steps that are image acquisition, image segmentation and rendering, feature extraction and feature qualification from image, and databases and data sharing for eventual ad hoc informatics analysis [4]. In this paper, we investigate the role of the integration of the contrast agent kinetic heterogeneity features derived from breast dynamic contrast-enhanced magnetic resonance imaging and clinical feature from patient medical records for predicting molecular subtypes. The computerized quantitative image analysis in this paper includes precise breast lesion segmentation, phenotype extraction and clinical symptom, molecular subtypes prediction modeling, and leave-one-case-out cross validation. 637 patients that are all confirmed by pathological examination from one institution are used for discovery and external validation.

The primary goal of this paper is to develop an automated DCE-MRI-based lesion recognition method to distinguish the four molecular subtypes, which is helpful for the consequential treatment plan decision.

This work goes a step further on the original lesion data other than the intratumoral and peritumoral segmentation of tumor reported in [7, 8], in which a specialist marked the boundary contour of the lesion manually. There are many personal prejudices on the location or boundary of the tumor in different specialists. Moreover, the image patches containing the lesions are used in the prediction model on the lesion and lesion background data [9]. An automated segmentation method in this paper is used to extract the precise boundary of tumor. The major difference in the current work is the integration of higher visual features and dynamic features on actual lesion area from a larger patient cohort and combining multiple classifiers for feature validation. This is different from Banaie et al.'s method [10] and Fan et al.'s method [11], in which kinetic feature, such as ktrans, kep parameters extracted from 26 patients, and texture features from 173 patients, are validated by a logical regression without features selection. The imbalance problem in these datasets is ignored using a single classifier as we know that the morbidity of different molecular subtypes is serious different. In this work, we use radiomics features to distinguish between full four molecular subtypes other than on partial classes as work on luminal A and B in [9], or work on luminal A and other types in [11] by deep learning. These fused features for four subtypes allow not only characterization of cancer morphology, but also depiction of heterogeneity between imaging phenotypes and molecular subtypes of breast cancer.

**Figure 1:**
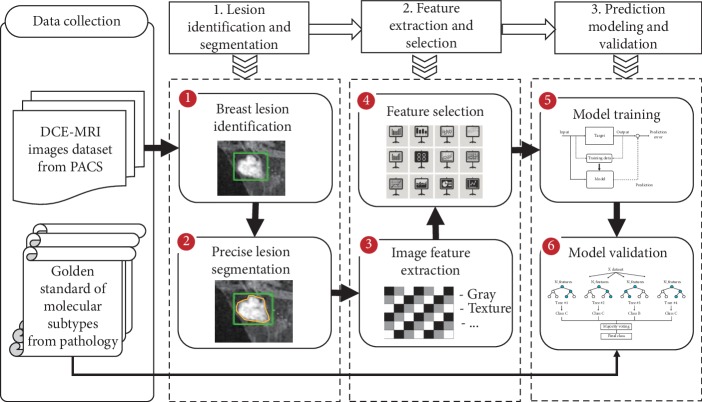
Workflow of presented breast cancer molecular subtypes recognition.

The workflow of the presented method is depicted in Figure 1. An improved region growth segmentation algorithm is applied on the lesion images. Different types of radiomics features are extracted from tumor data. Feature selection by a cascade validation method is conducted on both radiomics feature. A large patient cohort is collected from an institution, which is used for model training and testing. The main contributions of this work are as follows:

- An improved region growth algorithm with dynamic threshold setting is proposed on precise boundary of lesion segmentation, which not only saves time of automatic extraction of lesion region of interest without threshold setting for each case, but also prevents the segmentation error by manual and prejudice from different radiologists.
- The static visual features of texture, morphology, and statistics on lesion, dynamic kinetic features, and clinical features are extracted to validate the relationship between image phenotypes and the molecular subtypes, which is carried out on a largest patient cohort as we know from the latest work so far.
- The recursive feature elimination method based on multiple models is used to select useful features for prediction model, which pays attention to the imbalance problem of the dataset. The classification model based on DCE-MRI data achieves noninvasive molecular subtypes recognition, which improves the diagnostic efficiency of breast cancer.

The rest of this paper is organized as follows. In Section 2, we discuss previous related work.  Section 3 describes the details of the method. The experimental results and discussion are presented in Section 4, respectively. Finally, the concluding remarks are given in Section 5.

## 2. Related Work

The development of automated and reproducible analysis methodologies to extract more information from image-based features is a requirement [3]. Radiomics refers to the extraction and analysis of large amounts of advanced quantitative imaging features with high throughput from medical images, which leads to a very large potential subject pool [4]. Lots of visual features are extracted to quantify tumor image intensity, shape, and texture, which is associated with underlying gene-expression patterns [5, 6, 12, 13]. Combining with the medical character and clinical recognition of lung tumor, Wang et al. presented a radiomic analysis of 150 features to build a prediction model for malignant and benign discrimination of lung tumors [14]. It is also feasible to use radiomics approach to decode normal liver features and predict treatment-associated liver injury [15] and differentiate malignant nodules from benign ones [16].

DCE-MRI is one of the best imaging techniques that provide temporal information about the kinetics of the contrast agent, which is used to predict complete pathological response to neoadjuvant chemotherapy [7, 8, 17–19] and the risk of breast cancer recurrence in recent years [20–23]. Tumors exhibit genomic and phenotypic heterogeneity, which has prognostic significance and may influence response to therapy [1, 24]. Burgeoning genetic, epigenetic, and phenomenological data support the existence of intratumor genetic heterogeneity in breast cancers [2, 25, 26].

Banaie et al. proposed a method to help physicians determine the likelihood of malignancy in breast cancer using DCE-MRI without biopsy [10]. Quantitative radiomics of breast cancer may enable precision medicine with differentiating luminal A and luminal B breast cancer molecular subtypes [9, 27]. Three different deep learning approaches were used to classify the tumor according to their molecular subtypes. Computer-extracted image phenotypes as well as dynamic features from tumor and background parenchymal enhancement were used to determine DCE-MRI characteristics discriminating among four molecular subtypes of breast cancer [11, 28–31]. Deep learning with MRI dataset utilizing convolutional neural network may also play a role in discovering radiogenomic associations in breast cancer [32, 33].

The dataset used in this paper contains DCE-MRI image data and golden standard from pathology. A variety of radiomics features are extracted on the accurately segmented lesion data by an improved region growth algorithm and the automatic feature selection process is realized by recursive feature elimination optimization method, rather than manually selecting features. Secondly, the dataset contains a comprehensive range of molecular types, and the imbalance of each molecular subtype of data is considered in the predictive model, rather than considering small datasets and partial category recognition studies which are presented in existing research.

## 3. Methodology

The data collected from a hospital in this paper are all cases with malignant lesions confirmed by histopathology. Generally, the edge of the malignant lesion is not clear. It is difficult to extract the edge of the lesion area accurately because of the image background enhancement. However, it is difficult to fetch good characteristics for image phenotypes without accurate lesion area. Therefore, the approximate location of each lesion in this dataset is labeled by experienced radiologists, and it is a time-consuming work to annotate the area of the lesion. Meanwhile, the labeling results from different radiologists may be quite inconsistent. In this paper, the radiologists only marked out the lesion locations in the images. Then an improved regional growth algorithm is used to realize the automatic edge extraction of the lesions. Based on the extracted lesion regions, 142 image features including texture features, morphological features, statistical features, and dynamic enhancement characteristics are extracted. Feature selection is performed using the multimodel-based recursive feature elimination (mmRFE) method. The mmRFE method considers the sorting factors of each feature in each model other than the traditional RFE with single model. The models in mmRFE used in this paper are logistic regression (LR), support vector machine (SVM), random forest (RF), and gradient boosting decision tree (GBDT). Different classifiers differ in the recognition of molecular subtypes classification for patient cohort data which has imbalance problem on four molecular subtypes. The mmRFE method finds robust features for all four subtypes better than the classification effect of a single model in classification effect.

### 3.1. Lesion Segmentation

Breast lesions are relatively small. It will be useless if the radiomics features are extracted from the entire image. Therefore, it is general that the lesion areas are segmented firstly, on which the features are extracted.

There are generally three ways to extract lesions, automatic segmentation, manual segmentation, and interactive segmentation [34]. Automatic segmentation does not require human intervention, completely separated by the algorithms, that is also the focus of current research. However, this method is often inaccurate for complex image objects. Manual segmentation usually requires the assistance of an experienced operator, which is time-consuming and inaccurate for irregular images. Interactive segmentation firstly finds the approximate location of the region of interest (ROI) and marks it with a rectangular box, which has less human intervention and a good segmentation effect on complex images. This paper presents an interactive segmentation for breast lesions. The breast lesions are marked by two radiologists with 10 and 15 years experiences, respectively. The lesion in the ROI with border marks are connected areas and the grayscale is similar. It is known from above that the enhancement mode of breast lesion is mostly enhanced by internal interval, for which the regional growth (RG) algorithm has better segmentation effect.

The regional growth algorithm has two important influencing factors, namely, the selection of seed point and the definition of growth criteria. If the seed point is not selected properly, it is possible that the result of segmentation is very different from the original target and even the segmented result is wrong part of the image rather than the original target. As the lesions are labeled by the radiologists, the centroid of the ROI region is used as the seed point in this paper.

Once the seed point in target area is obtained, the surrounding connected pixels that follow the certain growth criteria are added to target areas one by one and finally complete the growth until there are no more connected pixels that follow the criteria.

**Figure 2:**
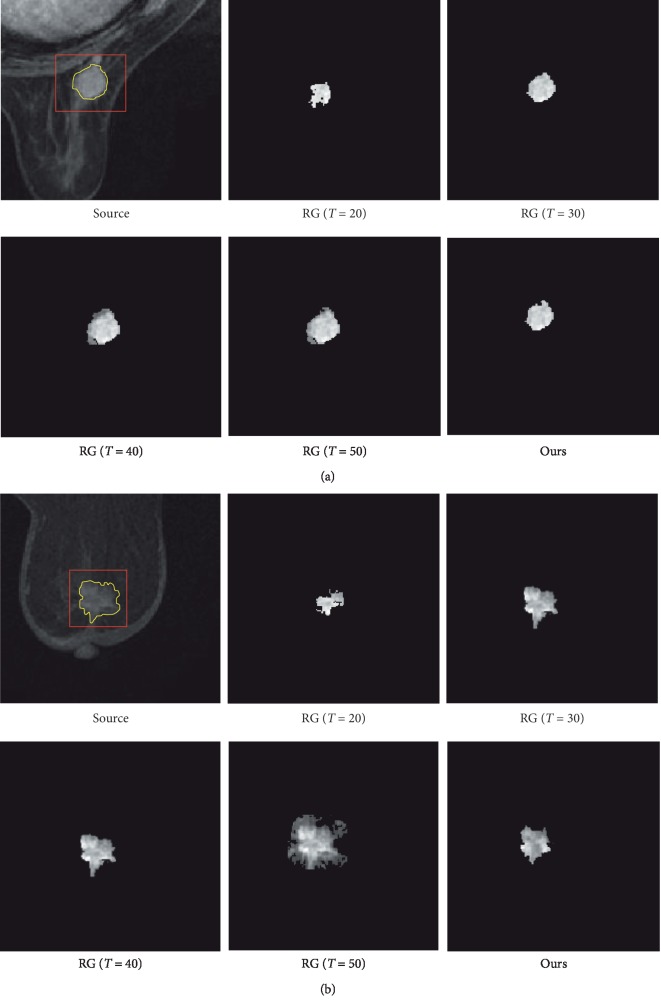
Breast cancer lesion segmentation. Regular lesion with smooth edge (a) and irregular lesion with more burrs (b). The lesion marked by rectangle and the actual border of lesion is shown as yellow curves; RG (*T* = 20, 30, 40, 50) shows the segmentation results by regular region growth algorithm. Ours is the result by the improved region growth algorithm in this paper.

The DCE-MRI images are grayscale images, so we only preset a certain threshold (*T*) that the pixel value is less than. Different growth thresholds have strictly different results on the segmentation effect of target results as shown in Figure 2 (*T*=20,30,40,50). The differences between the segmented results with different thresholds are obvious.

Figure 2 lists two types of lesion ROIs. The first in Figure 2(a) has a more regular shape, and the other ROI in Figure 2(b) is more irregular in shape besides more burrs. In this paper, the threshold value of segmentation growth is determined dynamically by the Otsu method, rather than by manual setting [35].

The results generated by our method are shown in Figure 2 (ours). Although the ROI in Figure 2(b) is more irregular and burr, the experimental result shows that the improved algorithm is still doing well. The improved regional growth algorithm not only reduces the artificial participation, but also saves the time, which makes the ROI segmentation more automated. The later feature extraction task is performed on precise lesions other than lesion with background which is generally used in exists works.

The lesion segmentation results are evaluated by the dice coefficient, which is a set similarity measurement function, as shown in formula (1). *X* represents the pixel set of the segmented lesion, and *Y* represents the actual collection of lesion pixels, where every pixel is represented as coordinate. The dice coefficient represents the percentage of the intersection of two sets that are segmented correctly. *S*=1 indicates that *X* and *Y* are fully coincident, and the segmentation accuracy rate is 100%. *S*=0 indicates that the segmentation results are totally wrong.

(1) $$\begin{array}{c} S=\frac{2\left|X\cap Y\right|}{\left|X\right|+\left|Y\right|}. \end{array}$$

In order to verify the accuracy of the lesion segmentation in this paper, the two lesions are manually hand-drawn by the radiologist to obtain the complete borders as shown in Figure 2 (source). The yellow curves are drawn by the radiologist manually. At the same time, the traditional region growth algorithm with different threshold and our method are conducted for comparison. It is seen that *t*=20 is obviously different from the lesion, and *T*=50 is obviously oversegmented. Therefore, the dice coefficients of the three thresholds (*T*=30,35,40) and our algorithm are evaluated, respectively, and the results are shown in Table 1.

As seen from the results of the evaluation indicators in Table 1, the traditional RG algorithm threshold cannot be determined automatically. It is necessary to find right segmentation threshold which is hard work for a large dataset. However, the results are greatly improved by our method, which dynamically searches the threshold without human interaction.

### 3.2. Feature Extraction

Once the lesions are segmented from DCE-MR images, the radiomics features are extracted consequently for molecular subtypes recognition, which is the quantitative expression of image information so that we can find effective imaging features. The effective features are important to realize the correct classification of breast cancer molecular subtypes. The breast cancer lesion is highly heterogenous. This characteristic presented in DCE-MRI images is quantified by textures in this paper. At the same time, the internal density of differences areas in lesion are changed over time and this feature is obtained by kinetics parameters.

The radiomics features including texture features, morphological features, statistical features, and kinetics features are designed in this paper.

#### 3.2.1. Texture Features

Texture reflects the arrangement properties of the surface organization of things, and it is a visual feature. Different tissues within the human body exhibit different textures in imaging examinations, and the same tissues exhibit different texture differences in a healthy area or in the lesion [36]. The image area has an invariant texture if a series of statistical or other characteristics of an image are fixed, slowly changing, or approximate [37, 38].

According to the characteristics of the lesion, the texture features of breast cancer were extracted by gray-level co-occurrence matrix (GLCM) and locality binary pattern (LBP), respectively.

- The GLCM is calculated from the pairs of pixel gray levels *i* and *j* that represent the probability of (*i*, *j*) appearing in a given spatial distance and direction, and all calculated results can be represented in the form of a matrix. This paper takes the direction as [0, 45, 90, 135]; that is, the GLCM is constructed in these four directions for the statistics characteristics of energy, entropy, deficit matrix, contrast, and correlation on three-time phase in each direction [39].
- LBP is an operator that characterizes local textures and is also used for texture feature extraction. The feature is then used in conjunction with the histogram of oriented gradient (HOG) feature classifier to improve the detection effect of some datasets [40–42]. The LBP mask used in this paper is the 3 × 3 matrix. If its value of each neighbor pixel is greater than the center point pixel value, the value of its location is set to 1. Otherwise, the center point pixel value is set to 0. This process will form a binary sequence with length 8, and then the value of the binary sequence as binary data is computed and is regarded as the LBP value. The computing process is shown as the formula (2) for a pixel (*x*, *y*), and *g*_*c*_ is the center pixel value and *g*_*p*_ is the neighbor pixel value.

(2) $$\begin{array}{c} \text{LBP}\left(x,y\right)=\overset{7}{\underset{p=0}{\sum }}S\left(g_{p}-g_{c}\right)*2^{p},\\ S\left(u\right)=\left\{\begin{array}{cc} 1, & u\geq 0,\\ 0, & u<0. \end{array}\right. \end{array}$$

(iii) The LBP matrix is computed by the formula applying all the pixels of the image, and then the histogram is extracted on the LBP matrix.

#### 3.2.2. Morphological Features

When a part of the tissue becomes a malignant lesion, it is usually accompanied by morphological changes. For example, the benign lesions of the breast are mostly lumpy, and the edges are smooth, while the malignant lesions are more morphological. Some malignant lesions are lumpy and the edges are irregular; others are diffuse with no obvious edge. The malignant tumor is surrounded by abundant blood vessels and has a strong aggression [43]. The BI-RADS standard divided the morphology of breast lesions into three types as mass, nonmass, and point-like [44]. The lumps are divided into circles, ellipses, and irregular shapes. The distribution of nonmass lesions is more diffuse and multiregional. The point-like lesions are usually less than 5 mm in diameter and are not easily detected displayed on enhanced images. The morphological features of breast DCE-MRI images in this paper mainly are designed as the morphological features in the study of breast molybdenum target images, which include standardized radial length mean and standard deviation, compactness, roughness, smoothness, roundness, and area [45].

#### 3.2.3. Kinetics Features

The dynamic enhancement characteristic presents the metabolism of the contrast agent in the lesion area which can provide the hemodynamic information of the lesion and shows the signal change of the lesion or normal tissue in different enhancement phase (8 phases in this paper) [46, 47]. The features are extracted on both the whole lesion and single pixel as study objects.

Firstly, the radiomics features extracted on the whole lesion includes lesion enhancement rate and absorption rate. The first phase in DCE-MRI is normal status without the contrast agent. The other phases are obtained where the lesion is enhanced that pixel's grayscales are relatively high. The lesion enhancement rate is expressed as

(3) $$\begin{array}{c} T=\frac{S_{i}}{S_{0}},\;i\in \left\{1,2\right\}, \end{array}$$

where *S*_*i*_ represents the grayscale mean of the pixels in lesion area of the corresponding time series. The enhancement rate reflects the aggregation degree of the contrast agent in the lesion. The absorption rate is expressed as formula (4), which represents the grayscale mean of the pixels in lesion area of the corresponding time series. The absorption rate of the lesion reflects the blood perfusion condition in the lesion.

(4) $$\begin{array}{c} T=\frac{\left(S_{i}-S_{0}\right)}{S_{0}}*100\%,\;i\in \left\{1,2\right\}. \end{array}$$

Secondly, the enhancement rate is defined on every pixel, which is expressed as

(5) $$\begin{array}{c} R_{Tt}=\left\{r\left|r\right.=\frac{I_{T}\left(i,j\right)-I_{t}\left(i,j\right)}{I_{t}\left(i,j\right)}\right\},\\ i=1,2,\ldots ,M,\\ j=1,2,\ldots ,N,\\ T\in \left\{1,2\right\},\\ i\in \left\{0,1\right\},\\ T>t, \end{array}$$

where *T* and *t* represent moments (such as *s*_0_, *s*_1_, *s*_2_ three-time phase), and the ROI matrix size is *M∗N*, *I*_*T*_(*i*, *j*) or *I*_*t*_(*i*, *j*) representing the pixel value of the *t* moment on image coordinate (*i*, *j*). The standard deviation, mean, and maximum dynamic characteristics are extracted using the obtained dataset.

#### 3.2.4. Statistics Features

The statistical characteristics of the image refer to the calculation of the grayscale values of each pixel point in the lesion. In this paper, the statistical features of three-time phase are extracted, including grayscale mean, standard deviation, information quantity, maximum value, peak degree, and deflection degree. Peak degree reflects the degree of steep easing of data distribution patterns. Deflection degree reflects the symmetry of the data distribution pattern.

Based on the three-time phase of breast cancer DCE-MRI images (three periods before and after adding contrast agents), the above paragraphs introduce the extraction of features, including texture, dynamics, statistics, and four types of morphological features. Among them, GLCM texture features include energy, contrast, correlation, entropy, and deficit matrix using representation as *F*_1_ ~ *F*_15_. LBP texture includes the three histograms as *F*_16_0 ~ *F*_16_255, *F*_17_0 ~ *F*_17_255, and *F*_18_0 ~ *F*_18_255. Dynamic characteristics include absorption rate, enhancement rate, standard deviation, mean, and maximum, represented as *T*_1_ ~ *T*_13_; statistical features include grayscale mean, grayscale standard deviation, information entropy, maximum value, deviation, and peak, labeled as *C*_1_ ~ *C*_18_. Morphological features include standardized radial length mean and standard deviation, tightness, roughness, smoothness, roundness, and area, known as *M*_1_ ~ *M*_7_. From the DCE-MRI sequential scans, we applied a computerized scheme to extract 142 imaging features while all invalid columns with 0 values are removed.  Table 2 summarizes these DCE-MRI features.

### 3.3. Prediction Model Training

The above feature extraction process generates a large number of radiomics feature data, but these features are not all useful for the recognition of molecular phenotypes. There are many methods of feature selection, and there is no strict uniform method of the feature selection for breast cancer DCE-MRI images. The feature selection is based on recursive feature elimination algorithm in this paper. The main idea of the recursive feature elimination (RFE) is to constantly repeat the build model, and each time, all features are sorted according to their importance. The least important features will be deleted until no more features can be deleted [48–50]. It can be seen that recursive feature elimination is a greedy algorithm.

Usually, a model is selected at first which is trained with sample data. The scores of importance for all features are calculated using the trained model, and the features with the least importance are removed from the current set of features. Then the remaining features are used in the model repeatedly until no features can be deleted. After the iteration is completed, the optimal feature subset is generated according to the evaluation criteria. The traditional recursive feature elimination is based on a single model for feature selection.

In the process of selecting features by the RFE method, the optimal subset of features selected by different classification models is varied. There is some overlap in the feature subsets for each model. In this paper, a multimodel-based recursive feature elimination (mmRFE) feature selection method is proposed. First, each model sorts all features according to their importance in order to get multiple sets of different sorts, and then the index of the positions of each feature in each set of sorts are recorded according to the sort results of each set of models. Finally, the index is summed up and the features are sorted again according to the sum results. A new comprehensive sort can be obtained. In the new sorted results, the index factors of each feature in different model are fully taken into account.

**Figure 3:**
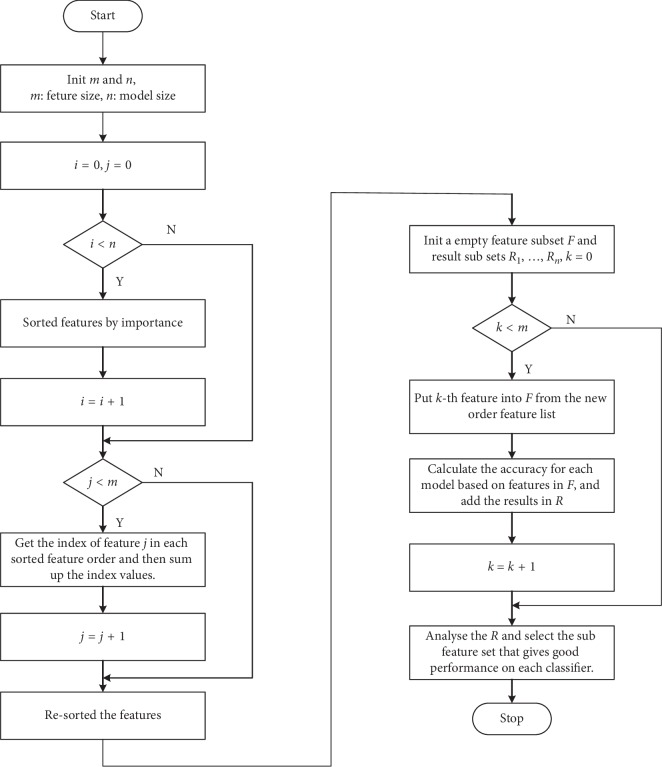
Flow chart of mmRFE algorithm for feature selection.

The comprehensive sorting features are used to train each model and the classification results are deposited into the result set. The lowest fractional features are removed by the importance of all the features in the comprehensive sort until no features can be deleted. Finally, each model will get multiple sets of results. Selecting a subset of features based on the results of each model makes this subset of features perform well in every model, such as a subset of features is selected that each classification model has an accuracy of more than 85%. The flow chart of the mmRFE method is shown in Figure 3.

The classification models to be trained in this paper include logistic regression (LR), support vector machine (SVM), random forest (RF), and gradient boosting decision tree (GBDT). The performance of each classifier is evaluated and discussed in the next section. The experimental results are obtained between traditional RFE based on single model and mmRFE in this paper.

## 4. Results and Discussion

### 4.1. Patient Population

In this paper, collected data of breast cancer DCE-MRI from a cancer hospital in Liaoning consist of 637 cases of patients in total. All 637 cases are malignant cases of breast cancer in women. The age range is between 43 and 70 years, and the average age is 57.2 ± 13.3 years.

These conditions are confirmed by histopathology examination after the patient received DCE-MRI examination which is diagnosed by radiologist. Diagnosis includes ductal carcinoma, invasive ductal carcinoma, invasive papillary carcinoma, mucous cancer, invasive lobular carcinoma, medullary carcinoma, solid papillary carcinoma, ductal carcinoma in situ, extensive ductal carcinoma, and extensive ductal carcinoma in situ. The pathological data of 637 patients are shown in Table 3 as well as the statistics of molecular subtypes. It is easy to see that the dataset has imbalance problem on molecular subtypes.

### 4.2. DCE-MRI Acquisition

The DCE-MRI data were generated by GE 1.5 T magnetic resonance imaging equipment (Hdx, GE Healthcare, waukesha, WI, USA) with breast dedicated 4-channel coil. Routine scanning parameters are axial T1WI SPGR sequence, sagittal T2WI fat inhibition sequence, and axial DWI sequence. The above sequence layer thickness is 3 mm, FOV for 36 *∗* 36 cm. DCE-MRI data take parameters as axial 3D dynamic SRGR sequence (TR 6.1, TE 2.9, Fov36 *∗* 36 cm, Matrix 512 *∗* 512) using the flip angle 2 degrees and 15 degrees scan to obtain T1 mapping, and then the flip angle 15 degrees for dynamic enhancement scanning. After collecting 1 phase sample, the high pressure syringe (Ulrich Medical) was injected intravenously Gd-DTPA 0.1 mmol/Kg, the injection rate was 3 ml/s, and the tube was washed with the 25 ml saline, and then the scanning of 8-time phase was continued.

### 4.3. Performance on Traditional RFE-Based Prediction Model

This paper uses four models LR, SVM, RF, and GBDT to select the optimal feature subset based on the traditional RFE with single model. The accuracy, precision, recall, and F1-score are used to evaluate classification performance.

The experimental results by LR show filtered features with 80 dimensions, including GLCM texture features with 9 dimensions (energy, contrast, correlation, deficit matrix in the first time phase, correlation in the second time phase, energy, correlation, entropy, and deficit matrix in the third time phase), morphological features with 2 dimensions (standardized radial length standard deviation, roughness), statistical features with 5 dimensions (the first phase of the grayscale standard deviation, the maximum grayscale, the second time phase of the grayscale mean, the maximum value, and the third time phase of the grayscale standard deviation), dynamic enhancement features with 7 dimensions (*T*_1,0_ standard deviation, mean value, maximum value, *T*_2,0_ mean, *T*_2,1_ standard deviation, mean, and maximum), and other LBP features.

The results from SVM experiment show that the features of the RFE filter are 77 dimensions, including the GLCM texture features with 8 dimensions (the contrast, correlation, deficit matrix of the first time phase, the correlation of the second time phase, the energy, contrast, entropy, and deficit matrix of the third time phase), and the morphological characteristics of 2 dimensions (standardized radial length mean and standard deviation), statistical features with 8 dimensions (grayscale mean, grayscale standard deviation, grayscale maximum, second time phase grayscale mean, bias, peak, third time phase grayscale standard deviation, and grayscale maximum), dynamic enhancement feature with 5 dimensions (*T*_1,0_ standard deviation, maximum value, *T*_2,0_ mean value, *T*_2,1_ the standard deviation, and the maximum value), and other LBP features.

The results of RF experiments show that the features of the RFE filter are a total of 55 dimensions, including GLCM texture features with 11 dimensions (energy, contrast, correlation, entropy, deficit matrix in the first phase, energy, contrast, correlation in the second phase, energy, contrast, correlation in the third time phase), morphological features with 4 dimensions (standardized radial length mean, standardized radial length standard deviation, tightness, roughness), statistical characteristics with 14 dimensions (first time phase grayscale mean, grayscale standard deviation, grayscale maximum, bias, peak, second time phase grayscale standard deviation, maximum value, bias, peak, third time phase grayscale mean, grayscale standard difference, grayscale maximum, bias, and peak), dynamic enhancement feature with 9 dimensions (*T*_1,0_ standard deviation, mean, maximum value, *T*_2,0_ standard deviation, mean value, maximum value, *T*_2,1_ standard deviation, mean value, and maximum value), and other LBP features.

The experimental results by GBDT show that the filtered features are 66 dimensions, including GLCM texture features with 13 dimensions (energy, contrast, correlation, deficit matrix in the first phase, energy, contrast, correlation, deficit matrix in the second time phase, energy, contrast, correlation, entropy in the third phase, and deficit matrix), morphological features with 4 dimensions (standardized radial length mean, standardized radial length standard deviation, tightness, roughness), statistical characteristics of with 14 dimensions (first time phase grayscale mean, grayscale standard deviation, bias, peak, second time phase grayscale mean, grayscale standard difference, maximum value, deviation, peak, grayscale mean, grayscale standard difference, grayscale maximum, deviation, and peak value of the third time phase), the dynamic enhancement feature with 8 dimensions (*T*_1,0_ standard deviation, mean, maximum value, *T*_2,0_ standard deviation, mean value, maximum value, *T*_2,1_ standard deviation, mean value), and other LBP features.

The feature subsets selected by the four models respectively are shown in Table 4, from which it is known that the subsets of features selected by the four classifiers are different.

As shown in Table 5, it can be seen from the experimental results that the GBDT has the best experimental results compared to the other models, which perform best in each evaluation index, followed by SVM and then RF, while the experimental results of LR is slightly worse, less than 0.8, and not as effective as the results of the remaining three models. If the molecular classification is based on the RFE single model, GBDT is best suited as the selected object.

### 4.4. Performance on mmRFE Based Prediction Model

In this experiment, the four classifiers are also used in RFE, respectively. The accuracy contained in each model is shown in Table 6. The logic regression accuracy is the lowest. Three feature subsets are found in all logistic regression experiments, in which the accuracy is more than 0.8. Compared with SVM, RF, and GBDT models, the first set for experimental results is more robust, so the first feature set is selected as the optimal subset of features in this experiment.

The selected feature subset with 69 dimensions includes GLCM texture features with 12 dimensions (energy, contrast, correlation, deficit matrix in the first phase, energy, correlation in the second phase, deficit matrix, energy, contrast, correlation, entropy, and deficit matrix in the third time phase), morphological features with 4 dimensions (standardized radial length mean, standardized radial length standard deviation, tightness, and roughness), statistical characteristics with 13 dimensions (first time phase grayscale mean, grayscale standard deviation, maximum value, second time phase grayscale mean, grayscale standard deviation, maximum value, bias, peak, third time grayscale mean, grayscale standard deviation, grayscale maximum, bias, and peak), and dynamic enhancement features with 6 dimensions (R10 mean, maximum value, R20 mean, R21 standard deviation, mean, and maximum), and the rest are LBP features. The detail features are *C*_14_, *T*_7_, *T*_11_, *T*_9_, *F*_17_247, *F*_18_243, *F*_16_15, *F*_5_, *F*_16_7, *F*_17_7, *T*_5_, *F*_11_, *F*_8_, *C*_4_, *C*_7_, *F*_16_248, *F*_18_245, *F*_16_11, *T*_13_, *F*_2_, *C*_12_, *C*_2_, *C*_11_, *M*_2_, *F*_13_, *F*_18_12, *T*_6_, *F*_17_12, *F*_17_242, *C*_16_, *F*_3_, *F*_1_, *C*_10_, *M*_1_, *F*_18_249, *F*_17_6, *T*_12_, *F*_16_4, *C*_1_, *F*_14_, *F*_18_15, *F*_12_, *F*_6_, *C*_17_, *F*_17_241, *F*_15_, *F*_18_9, *F*_17_246, *F*_18_250, *F*_17_10, *F*_18_244, *F*_18_252, *F*_17_5, *F*_16_245, *F*_10_, *C*_8_, *F*_17_240, *F*_18_14, *F*_16_14, *F*_16_250, *F*_18_1, *F*_16_246, *M*_4_, *F*_18_10, *F*_17_248, *C*_13_, *F*_18_7, *M*_3_, *F*_17_244 ordered by importance descendent.

The feature subset selected by the mmRFE is the result of considering the position factors of each feature in different models. The molecular subtypes classification is made by using the selected features and compared with the results selected by the single model. The validity of the selected features by the multimodel is further verified.

Based on the mmRFE, the feature screening is carried out by using the optimal feature subsets based on the current model selected by LR, SVM, RF, and GBDT, and the experimental results are displayed in combination with accuracy, precision, recall, and F1-score.

The performance evaluation on each molecular subtype classification by logistic regression is shown in Table 7, and it can be learnt from the table that the logistic regression has better classification performance on luminal A type and basal-like type.

The classification results by SVM are shown in Table 8 as well as the performance evaluation on each molecular subtype. The data in the table show that SVM has better classification effect of luminal A type, HER-2 expression type, and basal-like type of breast cancer. The luminal B type classification ability is weaker than the remaining three kinds.

The classification results by RF are shown in Table 9 as well as the performance evaluation on each molecular subtype. The data in the table show that RF has better classification effect of luminal A type, luminal B type, and HER-2 expression type of breast cancer. The basal-like type classification ability is weaker than the remaining three kinds.

The classification results by GBDT are shown in Table 10 as well as the performance evaluation on each molecular subtype. The data in the table show that GBDT has better classification effect on all types of breast cancers better than the above three classifiers.

From the results of each experiment, we can see that the identification ability of four classification models for the molecular classification of breast cancer is not identical and the three classification models LR, SVM, and RF cannot recognize the four molecular types of breast cancer very well that they are obviously weak for one or two subtypes of identification ability in molecular classification. GBDT is best suited as the selected classification model.

The four classification models are trained based on features selected by mmRFE, and classification results of each model are shown in Table 11. The performance of four classifiers is all good at stability especially for LR which behaves worst on feature selected by traditional RFE algorithm. In another words, the features selected by mmRFE algorithm are more optimal for molecular subtypes recognition task. The GBDT model obtains the best performance as well as good performance on the imbalance problem of molecular subtypes.

The results with different features and classier models are summarized in Table 12. From the experimental results, we can see that the experimental effect of the ensemble model classification using the features selected from multimodel RFE is better than that of each model using the features selected from the single model RFE method. Thus, it is proved that the multimodel feature selection method and the ensemble classifier are reasonable.

## 5. Conclusion

Breast cancer is a disease with high heterogeneity, and there are obvious differences in the response of different molecular subtypes to treatment. Therefore, recognizing molecular markers from DCE-MRI images directly to distinguish the four molecular subtypes without invasive biopsy is helpful for guiding treatment plans for breast cancer in early time. It will effectively improve the accuracy of breast cancer diagnosis and treatment from the breast DCE-MRI imaging phenotype, which reveals the quantitative imaging characterization mechanism of breast cancer molecular subtypes diagnosis, and improve the patient's five-year survival rate for grasping the treatment time. The current surgical biopsy is a pioneering, local tissue sampling. However, the use of DCE-MRI imaging that determines the molecular subtypes directly is noninvasive. This method can support comprehensive evaluation of heterogenecity of the lesions and predict the prognosis in advance.

This paper introduces an approach for molecular subtypes recognition and mainly focuses on the feature extraction and selection. In order to capture the precise feature description, the paper proposes an improved region growth algorithm to extract the precise edge of lesion based on radiologists' annotations. Then the various types of features of breast cancer phenotypes are extracted including texture, morphology, kinetic, and statistics features on different time phases of DCE-MRI. These features are not all useful for molecular subtypes recognition task. Therefore, the paper pays more attention to finding the best features. An mmRFE algorithm is proposed to select the feature subset, which is better than the traditional RFE algorithm based on the experimental results. Finally, we use the feature filtered by mmRFE algorithm to validate the performance of different classifier models as well as the imbalance performance of molecular subtypes on each model respectively. The GBDT obtains the best result on both classification and imbalance performance.

The future work will focus on extracting more features such as clinical features and the boost classification model. The problem should be discussed deeply in further work that strong model can find good features but bad for boost while weak models may be good in boost but cannot find useful features. The approach validated in treatment process will be another problem that should be also considered in the next work.

## Figures and Tables

**Table 1:** Evaluation result of image segmentation with different algorithms.

ID	Threshold	ROI (a)	ROI (b)	Mean dice
1	RG with *T*=30	0.712	0.652	0.682
2	RG with *T*=35	0.714	0.710	0.712
3	RG with *T*=40	0.622	0.632	0.627
4	Our method	0.897	0.877	0.887

**Table 2:** Summary of extracted radiomics features on DCE-MRI data.

ID	Features	Time phases	Detail features without 0 values	Feature labels
1	GLCM	*T*_0_	Energy, contrast, correlation, entropy, deficit matrix	*F*_1_ ~ *F*_5_
2	GLCM	*T*_1_	Energy, contrast, correlation, entropy, deficit matrix	*F*_6_ ~ *F*_10_
3	GLCM	*T*_2_	Energy, contrast, correlation, entropy, deficit matrix	*F*_11_ ~ *F*_15_
4	LBP	*T*_0_	Histogram index at [1, 2, 3, 4, 5, 6, 7, 8, 9, 10, 11, 12, 13, 14, 15, 240, 241, 242, 243, 244, 245, 246, 247, 248, 249, 250, 251, 252, 253, 254, 255]	*F*_16_0 ~ *F*_16_255
5	LBP	*T*_1_	Histogram index at [1, 2, 3, 4, 5, 6, 7, 8, 9, 10, 11, 12, 13, 14, 15, 240, 241, 242, 243, 244, 245, 246, 247, 248, 249, 250, 251, 252, 253, 254, 255]	*F*_17_0 ~ *F*_17_255
6	LBP	*T*_2_	Histogram index at [1, 2, 3, 4, 5, 6, 7, 8, 9, 10, 11, 12, 13, 14, 15, 240, 241, 242, 243, 244, 245, 246, 247, 248, 249, 250, 251, 252, 253, 254, 255]	*F*_18_0 ~ *F*_18_255
7	Kinetic	*T*_1,0_/*T*_2,0_/*T*_2,1_	Standard deviation, mean, maximum value	*T*_1_ ~ *T*_9_
8	Kinetic	*T*_1,0_/*T*_2,0_	Enhancement rate, absorption rate	*T*_10_ ~ *T*_13_
9	Statistics	*T*_0_	Grayscale mean, grayscale standard deviation, information entropy, grayscale maximum value, bias, peak	*C*_1_ ~ *C*_6_
10	Statistics	*T*_1_	Grayscale mean, grayscale standard deviation, information entropy, grayscale maximum value, bias, peak	*C*_7_ ~ *C*_12_
11	Statistics	*T*_2_	Grayscale mean, grayscale standard deviation, information entropy, grayscale maximum value, bias, peak	*C*_13_ ~ *C*_18_
12	Morphology	*T*_0_	Standardized radial length mean, standardized radial length standard deviation, tightness, roughness, smoothness, roundness, area	*M*_1_ ~ *M*_7_

**Table 3:** Patient cohort collection with pathological and molecular subtypes.

Pathology	Luminal A	Luminal B	HER-2	Basal-like	Total
Intracatheter cancer	6	16	8	4	34
Invasive ductal carcinoma	171	209	131	60	571
Invasive micropapillary carcinoma	0	6	2	0	8
Mucous carcinoma	0	2	0	0	2
Invasive lobular carcinoma	2	4	2	2	10
Medullary carcinoma	0	0	0	2	2
Solid papillary carcinoma	2	0	0	0	2
Ductal carcinoma in situ	0	2	0	2	4
Extensive ductal carcinoma	2	0	0	0	2
Extensive ductal carcinoma in situ	0	2	0	0	2
Total	183	241	143	70	637

**Table 4:** Summary of features selected by traditional RFE algorithm.

No.	Model	Features selected (sorted by importance descent)	Size
1	LR	*C*_10_, *C*_7_, *T*_13_, *T*_11_, *C*_14_, *F*_18_243, *F*_18_245, *F*_18_249, *F*_16_7, *F*_17_247, *F*_17_7, *F*_16_15, *F*_17_249, *F*_5_, *F*_11_, *F*_15_, *C*_4_, *T*_9_, *F*_9_, *F*_16_11, *T*_7_, *F*_16_254, *F*_16_4, *T*_5_, *F*_16_248, *F*_18_244, *F*_16_249, *F*_16_251, *F*_18_2, *F*_16_1, *F*_18_7, *F*_17_240, *F*_18_10, *F*_18_254, *F*_16_10, *F*_18_15, *F*_18_250, *F*_18_9, *F*_17_245, *F*_18_12, *F*_17_253, *F*_17_6, *F*_17_248, *F*_18_1, *F*_18_252, *F*_17_5, *F*_16_247, *F*_17_246, *F*_17_242, *F*_16_245, *F*_16_246, *F*_18_14, *F*_8_, *F*_13_, *F*_16_8, *F*_17_1, *F*_17_4, *F*_17_241, *F*_17_254, *F*_18_11, *F*_2_, *F*_16_243, *F*_18_253, *F*_17_12, *F*_17_10, *F*_18_6, *F*_17_250, *F*_16_240, *F*_1_, *F*_3_, *C*_2_, *F*_17_255, *F*_18_5, *F*_14_, *F*_16_3, *T*_6_, *T*_12_, *F*_16_250, *M*_4_, *M*_2_	80

2	SVM	*F*_18_245, *F*_16_15, *F*_17_247, *F*_18_243, *F*_16_7, *F*_17_7, *T*_13_, *T*_11_, *C*_14_, *T*_7_, *T*_9_, *C*_16_, *C*_4_, *F*_5_, *C*_7_, *F*_17_12, *T*_5_, *F*_18_248, *F*_17_248, *F*_16_11, *F*_16_248, *F*_14_, *F*_11_, *F*_16_1, *F*_18_244, *F*_17_240, *F*_18_10, *F*_15_, *F*_18_7, *M*_1_, *M*_2_, *F*_16_3, *F*_16_4, *F*_18_2, *F*_17_243, *F*_17_6, *F*_18_249, *F*_16_245, *F*_17_15, *F*_18_13, *C*_11_, *C*_12_, *F*_18_252, *F*_18_14, *F*_17_5, *F*_18_251, *F*_17_4, *F*_17_245, *F*_18_12, *F*_18_9, *F*_18_15, *F*_18_254, *F*_8_, *F*_16_254, *F*_18_3, *F*_18_250, *F*_18_255, *F*_17_242, *F*_16_6, *F*_17_3, *F*_17_10, *F*_17_9, *F*_17_241, *F*_16_246, *C*_2_, *F*_3_, *F*_2_, *F*_12_, *F*_16_251, *C*_1_, *F*_17_244, *F*_18_240, *F*_18_8, *F*_17_1, *F*_17_246, *F*_16_242, *F*_17_249	77

3	RF	*F*_1_, *C*_11_, *T*_7_, *F*_7_, *F*_17_247, *F*_13_, *C*_1_, *F*_8_, *F*_2_, *C*_2_, *C*_17_, *F*_16_15, *T*_9_, *F*_6_, *C*_14_, *C*_12_, *T*_6_, *C*_13_, *C*_4_, *F*_5_, *C*_5_, *T*_12_, *F*_17_8, *M*_3_, *F*_16_11, *F*_11_, *C*_10_, *F*_12_, *T*_11_, *C*_18_, *T*_5_, *F*_16_248, *F*_4_, *C*_8_, *F*_18_243, *M*_4_, *F*_16_250, *T*_13_, *M*_1_, *M*_2_, *T*_8_, *F*_18_12, *F*_18_6, *C*_6_, *T*_10_, *F*_3_, *F*_16_5, *F*_17_242, *F*_18_246, *F*_17_7, *F*_17_12, *F*_17_10, *F*_18_250, *F*_18_245, *C*_16_	55

4	GBDT	*F*_1_, *T*_7_, *C*_14_, *F*_3_, *F*_8_, *M*_1_, *C*_17_, *T*_6_, *C*_12_, *M*_3_, *F*_13_, *F*_16_7, *F*_10_, *F*_2_, *T*_11_, *T*_9_, *F*_16_250, *C*_2_, *C*_5_, *C*_16_, *M*_2_, *F*_16_14, *T*_12_, *F*_17_7, *F*_17_2, *F*_7_, *C*_6_, *T*_10_, *F*_17_242, *C*_18_, *C*_1_, *C*_11_, *F*_5_, *F*_18_243, *F*_17_244, *F*_16_245, *T*_5_, *M*_4_, *F*_17_13, *F*_12_, *F*_18_1, *F*_14_, *F*_6_, *F*_17_252, *F*_18_12, *F*_17_241, *C*_8_, *F*_11_, *F*_17_246, *C*_10_, *F*_17_12, *C*_13_, *F*_16_4, *F*_16_2, *F*_16_15, *F*_17_247, *F*_17_6, *F*_15_, *T*_8_, *F*_16_252, *F*_18_15, *C*_7_, *F*_18_9, *F*_18_4, *F*_18_246, *F*_16_248	66

**Table 5:** Performance evaluation of each model on its respective optimal feature subset.

No.	Classifier	Accuracy	Precision	Recall	F1-score
1	LR	0.79	0.79	0.79	0.78
2	SVM	0.86	0.88	0.85	0.86
3	RF	0.82	0.83	0.83	0.83
4	GBDT	0.88	0.89	0.87	0.88

**Table 6:** Accuracy of three feature subsets in each classification model.

No.	LR	SVM	RF	GBDT	Average	Feature size
1	0.8006	0.8105	0.8291	0.8559	0.8240	69
2	0.8005	0.7987	0.7864	0.8348	0.8051	77
3	0.8096	0.8087	0.7814	0.8479	0.8119	86

**Table 7:** Classification of molecular of LR.

Molecular subtype	Precision	Recall	F1-score
Luminal A	0.95	0.88	0.91
Luminal B	0.70	0.73	0.71
HER-2	0.67	0.79	0.73
Basal-like	0.94	0.84	0.89

**Table 8:** Classification of molecular of SVM.

Molecular subtype	Precision	Recall	F1-score
Luminal A	0.97	0.93	0.95
Luminal B	0.74	0.63	0.68
HER-2	0.80	0.87	0.83
Basal-like	0.85	0.97	0.91

**Table 9:** Classification of molecular of RF.

Molecular subtype	Precision	Recall	F1-score
Luminal A	0.94	0.91	0.92
Luminal B	0.86	0.93	0.89
HER-2	0.85	0.89	0.87
Basal-like	0.72	0.61	0.66

**Table 10:** Classification of molecular of GBDT.

Molecular subtype	Precision	Recall	F1-score
Luminal A	0.91	0.90	0.90
Luminal B	0.89	0.91	0.90
HER-2	0.83	0.82	0.82
Basal-like	0.87	0.83	0.85

**Table 11:** Comparison of classification results of each model on features selected by mmRFE.

Classifier	Accuracy	Precision	Recall	F1-score
LR	0.80	0.82	0.81	0.81
SVM	0.85	0.84	0.85	0.84
RF	0.83	0.84	0.84	0.84
GBDT	0.87	0.88	0.87	0.87

**Table 12:** Performance evaluation for all hypotheses discussed in this paper.

No.	Features	Size	Classifier	Accuracy	Precision	Recall	F1-score
1	RFE	80	LR	0.79	0.79	0.79	0.78
2	RFE	77	SVM	0.86	0.88	0.85	0.86
3	RFE	55	RF	0.82	0.83	0.83	0.83
4	RFE	66	GBDT	0.88	0.89	0.87	0.88
5	mmRFE	69	LR	0.80	0.82	0.81	0.81
6	mmRFE	69	SVM	0.85	0.84	0.85	0.84
7	mmRFE	69	RF	0.83	0.84	0.84	0.84
8	mmRFE	69	GBDT	0.87	0.88	0.87	0.87
9	mmRFE	69	Ensemble	0.90	0.89	0.90	0.90

## Data Availability

The patient population data used to support the findings of this study have not been made freely available because the data are supplied by the Cancer Hospital of Liaoning under license. Requests for access to these data should be made to the corresponding author.
